# Maternal blood manganese level and birth weight: a MOCEH birth cohort study

**DOI:** 10.1186/1476-069X-13-31

**Published:** 2014-04-29

**Authors:** Jin-Hee Eum, Hae-Kwan Cheong, Eun-Hee Ha, Mina Ha, Yangho Kim, Yun-Chul Hong, Hyesook Park, Namsoo Chang

**Affiliations:** 1 Department of Social and Preventive Medicine, Sungkyunkwan University School of Medicine, 2066 Seobu-ro, Jangan-gu, Suwon, Gyeonggi-do 440-746, Republic of Korea; 2 Department of Social and Preventive Medicine, Samsung Biomedical Research Institute, Sungkyunkwan University School of Medicine, 81 Irwon-ro, Gangnam-gu, Seoul 135-990, Republic of Korea; 3 Department of Preventive Medicine, Ewha Womans University School of Medicine, 1071 Anyangcheon-ro, Yangcheon-gu, Seoul 158-710, Republic of Korea; 4 Department of Preventive Medicine, Dankook University College of Medicine, 119 Dandae-ro, Dongnam-gu, Cheonan, Chungnam 330-714, Republic of Korea; 5 Department of Occupational and Environmental Medicine, University of Ulsan College of Medicine, 877 Bangeojinsunhwando-ro, Dong-gu, Ulsan 682-714, Republic of Korea; 6 Department of Preventive Medicine, Seoul National University College of Medicine, 103 Daehak-ro, Jongno-gu, Seoul 110-799, Republic of Korea; 7 Department of Nutritional Science and Food Management, Ewha Womans University, 52 Ewhayeodae-gil, Seodaemun-gu, Seoul 120-750, Republic of Korea

**Keywords:** *In utero* environment, Birth outcome, Birth cohort, Foetal development, Dose–response relationship, Manganese

## Abstract

**Background:**

Manganese (Mn) is an essential trace element for humans and animals, but excess intake of Mn can lead to adverse developmental outcome. Few studies have investigated the effects of deficiency or excess of Mn on the human foetus. In this study, we assessed the quantitative relationship between maternal blood Mn and birth weight of a newborn.

**Methods:**

We performed analysis on 331 full-term, live birth singleton mother-infant pairs enrolled from July 2007 to December 2009 in the Mother and Children’s Environmental Health (MOCEH) study in Korea. A questionnaire on general characteristics, a review of medical records, and maternal whole blood Mn analysis were performed at full-term pregnancy. We evaluated the relationship between maternal blood level of Mn and the birth outcome using logistic regression and generalised additive model.

**Results:**

The mean Mn concentration in whole maternal blood was 22.5 μg/L. We found a curvilinear relationship between maternal blood Mn and birth weight after adjusting for potential confounders. Birth weight peaked at the maternal blood Mn level of 30 and 35 μg/L. An increased probability of birth weight below 3000 g was observed at both below 16.9 μg/L (odds ratio = 2.77, 95% CI: 0.89–8.65) and above 26.9 μg/L of maternal blood Mn level (odds ratio = 2.66, 95% CI: 0.84–8.08).

**Conclusions:**

Our study found that both extreme level of maternal Mn level was associated with lower birth weight outcome in a nonlinear fashion.

## Introduction

Manganese (Mn) is one of the essential nutrients for humans and animals [1]. It is required for energy metabolism, development of the skeletal system, activation of certain enzymes, function of reproductive hormones, and antioxidant functions that protect cells [1,2]. Mn deficiency may result in poor bone formation, birth defects, and increased susceptibility to seizures [3-5]. Such a health outcome is, however, rarely reported in humans because the element is widely present in common foods [6].

Pregnant women and infants typically show an increase in blood Mn level, which becomes more prominent in the later phase of pregnancy [7]. High Mn demands of the developing foetus during pregnancy lead to increased blood Mn level. Foetuses and neonates could be at higher risk for the toxic effects of high Mn exposure because they do not have fully developed homeostatic mechanisms for Mn [8]. Generally, adults maintain stable blood Mn concentrations by Mn homeostasis, which is achieved by regulation of absorption and excretion [1]. Studies demonstrate increased gastrointestinal absorption of Mn [9] and decreased ability to eliminate Mn [10,11].

Mn-related maternal and developmental toxicities have been observed in studies of experimental animals, including reduced foetal body weight and high Mn level [12,13]. Few epidemiologic studies reported the relationship between maternal blood Mn level and birth weight of pregnancy outcome in human [14,15]. In a study in Teheran, Iran, intrauterine growth retardation was linearly associated with lower maternal blood Mn level, but with higher cord blood Mn level [14]. In another study, birth weight was biphasically associated with maternal blood Mn concentration in an inverted U-shaped dose–response relationship [15]. This study was conducted in a population living near a lead and zinc mining site in northeastern Oklahoma, U.S.A., with a potential environmental metal exposure. There are few reports on the birth outcome of Mn level during pregnancy in the general population [16]. Also, the effect of the very low level of Mn was less explored. Little is known about the effects of deficiency or excess of Mn on infant growth or birth outcome in humans [16]. Uncertainty still remains about the degree which Mn level will be adequate for pregnant woman. The objective of this study was to assess the association between maternal blood Mn concentrations during pregnancy and birth weight in the general population without a prominent source of Mn exposure.

## Methods

### Study subjects

This analysis was conducted on a community-based birth cohort study, the Mothers and Children’s Environmental Health (MOCEH) study. MOCEH is designed to investigate the effects of pregnant women’s environmental exposure on foetal and postnatal growth and development in three centres; Seoul, Cheonan, and Ulsan, Republic of Korea [17]. Among 953 participants recruited until 2009, research committee of MOCEH approved blood sampling for Mn on 352 pregnant women consecutively recruited between July 2007 and December 2009 from three centres. Study subjects agreed to undergo maternal blood Mn analysis and had available birth records. For the analysis, we excluded stillborn baby (n = 1), pregnancy-related diabetes (N = 3), severe foetal stress (N = 4), twins (n = 4), and preterm (<37 weeks, n = 8) and over-term (≥42 weeks, n = 1) infants, leaving 331 mother-infant pairs as study subjects.

This study protocol was approved by the institutional review boards of Ewha Womans University, Dankook University Hospital, and Ulsan University Hospital. Informed consent was obtained from all the study participants at the time of recruitment.

### Methods

Data were collected by a questionnaire and from each woman’s medical records before 20 weeks of gestation by a trained nurse at the outpatient clinic and at the time of visit for delivery. The questionnaire consisted of socio-demographic information such as mailing address, age, height, weight, occupation, education, and income; pre-pregnancy alcohol consumption; smoking history; complications of the current pregnancy; and the subject’s biological, medical, and obstetric history. At delivery, trained nurses measured birth weight using a digital scale.

Medical records were reviewed to collect information on maternal pre-pregnancy weight, maternal height, gestational age, parity information, delivery mode, infant sex and birth weight, presence of preterm birth or low birth weight, delivery date, maternal term weight and presence of intrapartum complications including premature rupture of membranes, cephalopelvic disproportion, dystocia, breech presentation or other abnormal position, placenta abruption, placenta previa, meconium aspiration syndrome, foetal asphyxia, obstetrical haemorrhage, precipitating labour, cord prolapse or eclampsia during labour, fever, and use of anaesthetics.

### Blood collection

Maternal venous blood samples (10 ml) were obtained at the visit for delivery and stored at -70°C until it was transferred to the laboratory at the Neodin Medical Institute for analysis. Whole blood Mn level was measured by graphite furnace atomic absorption spectrophotometry (AAnalyst 600, PerkinElmer, Waltham, MA, USA). The laboratory analyses were conducted using standardised quality-control procedures from the German External Quality Assessment Scheme and Institute and Outpatient Clinic for Occupational, Social and Environmental Medicine of the University of Erlangen-Nuremberg as described by Kim *et al*. [17]. The relative standard deviation of the analysis was below 5%. The limit of detection for Mn was 1.585 μg/L and all samples were above the limit of detection.

### Data analysis

The distribution of maternal blood Mn was slightly skewed to the right (skewness = 1.3), but the distribution was close to Gaussian except for outliers above the 99th percentile value. Therefore, we did not transform it to a log scale. Student’s t-test or ANOVA was performed for unadjusted group differences in maternal blood Mn and birth weight. We assessed the association between maternal Mn level and birth weight by simple regression analysis and then by multiple regression analysis using a second degree polynomial function after adjusting for potential confounders (infant sex, gestational age, maternal education, maternal parity, maternal term weight, maternal income, and maternal age), which were determined from previous studies [6,14,15,18,19].

Multiple logistic regression was used to assess the relationship between maternal blood Mn and the birth outcome after adjusting for the potential confounders. We categorised the birth weight variable as binary data: lower than 3000 g (which represented less than 20 percentile among the study population) or equal or more than 3000 g. Non-linear relationships were examined by a generalised additive model (GAM) after controlling for potential confounders. Statistical analyses were performed using SAS 9.3 software (SAS Institute Inc., Cary, NC, USA) and R software (R version 3.0.1). Statistical significance was determined using a *p* value of < 0.05.

## Results

The arithmetic mean (SD) of blood Mn concentration in this study population was 22.5 (7.2) μg/L, ranging from 8.5 to 58.6 μg/L, and the median was 21.5 μg/L. Mean infant birth weight was 3303.9 (367.6) g, and mean maternal age was 30.3 (3.6) years. The gestational age and maternal term weight were significantly associated with birth weight, but only gestational age was associated with maternal blood Mn in borderline level of significance (p = 0.067) (Table 1).

**Table 1:** Distribution of blood manganese concentration and birth weight by general characteristics of the study subjects

			**Maternal blood Mn (μg/L)**		**Birth weight (g)**	
	**N**	**(%)**	**Mean ± SD**	**p-value**^**†**^	**Mean ± SD**	**p-value**^**†**^
Total	331	(100)	22.5 ± 7.2	-	3,303.9 ± 367.6	-
Infant sex						
Male	166	(50.2)	22.7 ± 7.5	0.521	3,359.2 ± 388.7	0.004
Female	160	(48.3)	22.2 ± 6.5		3,242.6 ± 331.6	
Missing	5	(1.5)	25.4 ± 15.1	-	4060.0	-
Gestational age (days)						
<268	79	(23.9)	20.9 ± 8.1	0.067	3,252.4 ± 363.6	<.001
268-272	73	(22.0)	22.9 ± 6.7		3,169.5 ± 333.6	
273-277	54	(16.3)	22.8 ± 7.1		3,292.2 ± 346.0	
278-282	67	(20.2)	23.1 ± 6.7		3,447.4 ± 385.8	
≥283	58	(17.5)	23.2 ± 7.0		3,389.8 ± 346.2	
Maternal age (years)						
<28	104	(31.4)	22.5 ± 7.6	0.781	3,312.6 ± 343.7	0.996
28-29	90	(27.2)	21.1 ± 6.8		3,282.2 ± 395.9	
30-32	82	(24.8)	22.8 ± 6.7		3,326.3 ± 372.3	
≥33	54	(16.3)	22.6 ± 7.8		3,296.4 ± 364.0	
Missing	1	(0.3)	29.8	-	2,920.0	-
Maternal education						
High school or less	85	(25.7)	22.7 ± 7.3	0.794	3,337.5 ± 398.7	0.400
University or above	230	(69.5)	22.5 ± 7.1		3,301.1 ± 359.7	
Missing	16	(4.8)	21.8 ± 8.2	-	3,166.9 ± 284.6	-
Maternal income (million KRW^‡^ per month)						
<200	81	(24.5)	22.8 ± 6.4	0.961	3,280.6 ± 391.4	0.276
200-299	117	(35.3)	21.8 ± 7.3		3,295.7 ± 375.6	
≥300	118	(35.6)	22.9 ± 7.2		3,336.5 ± 349.7	
Missing	15	(4.5)	22.3 ± 9.7	-	3,233.6 ± 315.7	-
Maternal parity						
No	134	(40.5)	22.3 ± 6.4	0.363	3,281.9 ± 358.9	0.821
≥1	151	(45.6)	22.4 ± 7.9		3,334.4 ± 370.5	
Missing	46	(13.9)	23.5 ± 6.9		3,265.0 ± 382.0	
Maternal term weight (kg)						
<61	54	(16.3)	24.4 ± 8.5	0.736	3,182.4 ± 333.6	0.003
61-64	56	(16.9)	21.6 ± 7.0		3,244.6 ± 283.0	
65-68	36	(10.9)	21.6 ± 6.5		3,251.4 ± 383.9	
69-75	50	(15.1)	21.5 ± 4.5		3,368.4 ± 358.0	
≥76	43	(13.0)	21.7 ± 7.8		3,519.3 ± 426.7	
Missing	92	(27.8)	23.2 ± 7.4		3,295.6 ± 361.2	
Maternal blood Mn (μg/L)^*^						
<16.9	65	(19.6)	-	-	3,362.2 ± 386.6	0.167
16.9 – <20.0	66	(19.9)	-	-	3,225.3 ± 378.5	
20.0 – <22.9	68	(20.5)	-	-	3,355.5 ± 345.3	
22.9 – <26.9	66	(19.9)	-	-	3,270.2 ± 347.9	
>26.9	66	(19.9)	-	-	3,308.6 ± 371.5	
Birth weight (quintile, g)						
<3000	59	(18.2)	22.7 ± 8.6	0.608	-	-
3000-3199	65	(20.0)	21.5 ± 6.1		-	-
3200-3399	66	(20.3)	21.8 ± 6.0		-	-
3400-3599	64	(19.7)	22.8 ± 7.1		-	-
≥3600	71	(21.9)	23.2 ± 7.4		-	-

^†^Tested by ANOVA (tested for linear trend in case of ordinal variables) or t-test.

^‡^10^6^ KRW = 1050 USD as of Jan 2008.

^*^The 8 μg/L unit categories of blood Mn.

(N = 331).

When we evaluated the quadratic association of Mn on birth outcome using a multiple linear regression model, a borderline level of significance was found between blood Mn and birth weight (p = 0.054) after controlling for possible confounders including infant sex, gestational age, maternal education, maternal parity, maternal term weight, maternal income, and maternal age (Table 2).

**Table 2:** Regression analysis of birth weight and maternal blood Mn concentration

	**Model 1**^**‡**^			**Model 2**^**†**^			**Model 3**^**†**^		
	**Beta**	**SE**	***p*-value**	**Beta**	**SE**	***p*-value**	**Beta**	**SE**	***p*-value**
Maternal Mn	3.39	2.90	0.244	2.65	2.87	0.356	23.48	12.14	0.054
Maternal Mn^2^	-	-	-	-	-	-	-0.38	0.22	0.079
Model *p* value	0.244			<0.001			<0.001		
Adjusted R^2^	0.001			0.163			0.169		

^‡^Unadjusted model, ^†^Adjusted for infant sex, gestational age, maternal education, maternal parity, maternal term weight, maternal income, and maternal age (N = 325).

Table 3 shows the association between maternal blood Mn and infant birth weight. Multiple logistic regression analysis was performed to assess the relationship between maternal Mn level and binary categorised birth weight (below 3000 g or more than 3000 g) after categorising the Mn level into five levels. Both lowest (<16.9 μg/L) and highest (≥26.9 μg/L) categories of maternal blood Mn levels were associated with birth weight below 3000 g infants; the odds ratio of the former was 2.77 (95% CI: 0.89 – 8.65) and the latter was 2.60 (95% CI: 0.84 – 8.08), although it was significant at borderline level (p = 0.079 and p = 0.098, respectively).

**Table 3:** Logistic regression analysis of birth weight below 3000 g and maternal blood Mn concentration

**Maternal blood manganese level (μg/L)**	**No. of subjects**	**AOR**^**†**^	**95% confidence interval**	***p*-value**
<16.9	66	2.77	(0.89-8.65)	0.079
16.9 – <20.0	65	1.41	(0.42-4.79)	0.580
20.0 – <22.9	65	2.43	(0.79-7.52)	0.123
22.9 – <26.9	65	1.00	-	-
>26.9	65	2.60	(0.84-8.08)	0.098

^†^Adjusted odds ratio, adjusted for infant sex, gestational age, maternal education, maternal parity, maternal term weight, maternal income, and maternal age.

**Figure 1:**
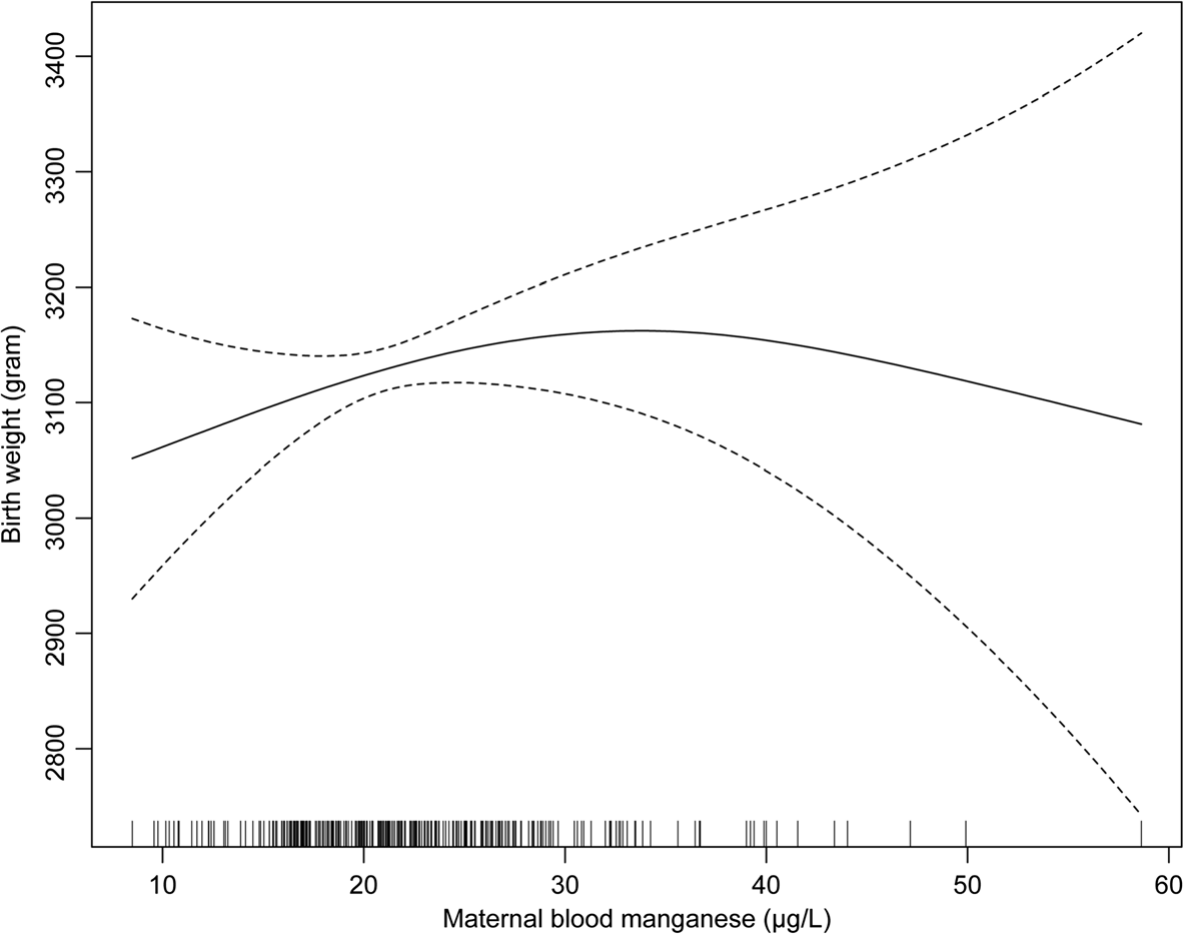
**Nonlinear association between birth weight and maternal blood Mn level. **^†^adjusted for infant sex, gestational age, maternal education, maternal parity, maternal term weight, maternal income, and maternal age.

We obtained consistent results with the generalised additive model, showing that low and high levels of Mn were associated with birth weight below 3000 g. The birth weight gradually increased up to level of 30 μg/L Mn, then after a peak between 30 and 35 μg/L, decreased at over 35 μg/L level (Figure 1).

## Discussion

The results of our multicentre cohort study indicated that both low and high blood Mn level of pregnant women is associated with birth weight below 3000 g of infants. We further identified a curvilinear relationship between maternal blood Mn level and infant birth weight, in accordance with previous report [14].

It shows that a high level of blood Mn is associated with lower birth weight in full-term infants. A high maternal Mn level, over 36 μg/L, was borderline significantly associated with birth weight below 3000 g although the relationship between high Mn level and lower birth weight in our study population was rather weak. The report of Zota *et al.*[15] found a consistent relationship between blood Mn and birth weight in the area of the Tar Creek Superfund site, an area contaminated with mining waste. They reported an inverted U-shaped relationship with a concentration of 31 μg/L as the point of inflection between maternal blood Mn and birth weight. However, the mean concentration of maternal whole blood Mn was 2.4 μg/dL, which is higher compared to the level of our study population, and this difference was especially evident at the 95th percentile (4.1 μg/dL compared with 3.6 μg/dL in our study, data not shown), presumably because of potential environmental exposure related with the closed mine. Recent two China studies observed an inverted U-shaped relationship of maternal Mn and cord blood Mn with birth weight. In Shanghai study [20], they found that maternal Mn was associated with birth weight (n = 175, mean of maternal whole blood Mn = 6.6 μg/dL, mean of umbilical cord blood Mn = 8.5 μg/dL). In Dalian city study [21], northern China, significant relationship was observed between cord blood Mn and birth weight (n = 125, mean of maternal whole blood Mn = 55.0 μg/L, mean of umbilical cord blood Mn = 78.8 μg/L). The former study found that the birth weight increased Mn levels up to 4.18 μg/dL, the peak point of the latter study was 90.0 μg/L, and then decreased. The Mn concentrations of these studies were higher than other studies [14,15,22].

Another study conducted in Southwest Quebec also tested the hypothesis of an inverted U-shaped relationship, but did not obtain a significant association between maternal blood Mn and birth outcome. This study had a small sample size (n = 101) and the maternal Mn level (16.3 μg/L) was much lower than that in our study [22]. In Shanghai study [23], serum Mn from both cord blood and maternal blood were used as biomarkers, with the median maternal and cord serum level of 2.8 μg/dL and 4.0 μg/dL, respectively. The authors examined the relationship between cord serum Mn and birth weight by multiple regression, and did not find a significant association between them, although they found a negative relationship and nonlinear relationship between cord serum Mn and birth length and Ponderal index, respectively [23]. Because blood Mn level in the cord blood is higher than that in maternal blood at the time of delivery [20,21] and this study was conducted with serum, it cannot be directly comparable to our study.

Many studies reported the effects of Mn deficiency in animals, but human study is rare [3] to our knowledge. This may reflect inadequacy of data from developing countries or a lack of data on deficiency of Mn during pregnancy in developed countries. The most common features of Mn deficiency in animals were skeletal malformation, and deprivation of Mn intake results in abnormal glucose tolerance and perturbation of lipid and carbohydrate metabolism [24-28].

This study has a relatively small number of observations, especially at high Mn concentration range, which obscures the relationship at very high levels of maternal blood Mn. In addition, as we did not examine the association between umbilical cord blood Mn levels and birth weight, and maternal blood sampling during pregnancy was not measured. Thus, the temporal relationship between Mn level and birth outcome is unclear. We could not take the point that iron and Mn share uptake mechanisms into account, because the information on the maternal intake of nutritional supplement including iron and calcium, and iron-related health problems including anaemia were not available and prevalence of anaemia was very low. A study conducted in Sweden indicates that Mn level during pregnancy was not related to iron status [29].

Our results confirm the non-linear relationship reported in a previous human study [15] and the association between Mn exposure and lower birth weight in animal studies [30] even though our study was conducted on a female population without any specific environmental or occupational source of exposure.

## Conclusions

Our study found that both extreme level of maternal Mn level was associated with lower birth weight outcome in a nonlinear fashion.

## Abbreviations

Mn: Manganese; GAM: Generalised additive model.

## Competing interests

The authors declare that they have no competing interests.

## Authors’ contributions

JHE analysed the data and prepared the manuscript. HKC designed the study, supervised data analysis and preparation of the manuscript. EHH designed the study and managed the MOCEH project. Supervised the birth outcome data and approved the manuscript. MH conducted and managed the data from Cheonan Center of MOCEH. Supervised the data analysis and approved the manuscript. YK conducted and managed the data from Ulsan Center of MOCEH. Supervised the data analysis and approved the manuscript. YCH conducted the quality control of the blood sample analysis, supervised the data analysis, and approved the manuscript. HSP conducted and managed the data from Seoul Center of MOCEH. Supervised the data analysis and approved the manuscript. NSC conducted and approved the nutritional data and supervised the data analysis. All authors read and approved the final manuscript.
